# Development of a Taiwan cancer-related fatigue cognition questionnaire: reliability and validity

**DOI:** 10.18632/oncotarget.16285

**Published:** 2017-03-16

**Authors:** Shih-Chiung Lai, Wei-Chun Lin, Chien-Hsin Chen, Szu-Yuan Wu

**Affiliations:** ^1^ Department of Exercise and Health Science, National Taipei University of Nursing and Health Sciences, Taipei, Taiwan; ^2^ Department of Cancer Research Center, Wan Fang Hospital, Taipei Medical University, Taiwan; ^3^ Department of Colorectal Surgery, Wan Fang Hospital, Taipei Medical University, Taipei, Taiwan; ^4^ Institute of Toxicology, College of Medicine, National Taiwan University, Taipei, Taiwan; ^5^ Department of Radiation Oncology, Wan Fang Hospital, Taipei Medical University, Taipei, Taiwan; ^6^ Department of Internal Medicine, School of Medicine, College of Medicine, Taipei Medical University, Taipei, Taiwan; ^7^ Department of Biotechnology, Hungkuang University, Taichung, Taiwan

**Keywords:** cancer-related fatigue questionnaire, reliability, validity

## Abstract

**Purpose:**

We prospectively designed a Taiwan cancer-related fatigue cognition questionnaire, version 1.0 (TCRFCQ-V1.0), for Taiwanese patients with cancer and investigated the reliability and validity of this questionnaire.

**Results:**

The completion rate of the TCRFCQ-V1.0 was high (97% of the patients completed all items), and the rate of missing data was low (0.2%–1.1% for each item). Moreover, the Cronbach alpha value was 0.889. We eliminated 5 items because their respective Cronbach alpha values were higher than the total mean value of Cronbach's alpha. Overall, the TCRFCQ-V1.0 had adequate Cronbach alpha coefficients (range, from 0.882 to 0.889). In addition, the results of Bartlett's test were significant (chi-squared, 2390.11; *p* < 0.001), indicating the appropriateness of factor analysis. Sampling adequacy was confirmed by the Kaiser–Meyer–Olkin statistic of 0.868. Through exploratory factor analysis, we identified 6 factors with eigenvalues of > 1, and the scree plot indicated no flattening factors. Overall, 28 items achieved a factor loading of ≥ 0.55.

**Materials and Methods:**

We enrolled patients with cancer who were aged > 18 years, had received a pathological diagnosis of cancer, and had undergone cancer treatments such as surgery, chemotherapy, radiotherapy, or concurrent chemoradiotherapy at a single institute in Taiwan. Of the identified 167 eligible patients, 161 (96.4%) were approached. Of these patients, 6 (7.2%) declined to participate and 155 (92.8%) were interviewed. The initial 43 items in the TCRFCQ-V1.0 were assessed for ceiling and floor effects.

**Conclusions:**

The TCRFCQ-V1.0 is a reliable and valid instrument for measuring CRF cognition in Taiwanese patients with cancer.

## INTRODUCTION

Cancer-related fatigue (CRF) is a common, distressing, and potentially treatable condition [1]. Although CRF most commonly occurs during active cancer therapy, it may affect patients long after the completion of cancer treatment [2, 3]. Cancer patients with CRF could feel extremely tired in community activities, social events, relationshipd, and daily activities [4–6]. Friendship and family time might be loss because patients would spend more time sleeping which may cause school or work miss. Patients with CRF also could lead to mood changes and mental fatigue in some status [7]. CRF can cause cancer patients absent minded, think confused, and forget things easily. Financial problems might be encountered by CRF patients who leave from the jobs or cannot keep working full time. Taken together, CRF could reduce patients’ quality of life (QOL) and cause them to receive suboptimal cancer treatment [8].

Specific diagnostic criteria have been proposed for defining CRF as an independent entity in the 10th revision International Classification of Diseases (ICD-10) [9]. At least 6 of the 11 criteria must be met for establishing a diagnosis of CRF. In studies including diverse patient populations, approximately 10%–26% of patients were diagnosed as having CRF by using this definition [10]. However, the formal diagnostic criteria outlined in ICD-10 are not widely used, and patients are not necessarily required to meet a minimum number of criteria to receive a clinical diagnosis of CRF. Moreover, guidelines from expert groups, including the National Comprehensive Cancer Network (NCCN), concerning the screening and assessment of CRF do not recommend using the diagnostic criteria outlined in ICD-10 [1, 11, 12].

In Taiwanese cancer patients after cancer therapy, CRF is one of the most popular and suffering symptoms reported in Taiwan. A trusty and effective instrument for measuring CRF and fatigue-related cognition is essential for successful clinical care of Taiwanese patients with cancer who experience CRF. In this study, we prospectively designed a Taiwan CRF cognition questionnaire, version 1.0 (TCRFCQ-V1.0), for Taiwanese patients with cancer and investigated the reliability and validity of this questionnaire.

## RESULTS

### Patient characteristics

Of the identified 167 eligible patients, 161 (96.4%) were approached. Of these, 6 declined to participate. Thus, we interviewed 155 (92.8%) patients. We did not approach some eligible patients because they were discharged early, had poor communication skills and cognitive impairment, or were missed planned outpatient clinical visit. Three participants were later considered ineligible because they didn't receive cancer therapy, causing a study sample of 148 patients finally. Most of the patients were aged 40–59 years (*n* = 84, 57%), were men (*n* = 95, 64%), and had high school education (*n* = 62, 42%) or higher (*n* = 66, 45%). We collected most eligible cancer patients in our outpatient cancer clinics(*n* = 87, 59%). Furthermore, 31%, 19%, and 89% of the patients received a newly diagnosis of cancer within 3 months, had diagnosis of distant metastases, and were recently under adjuvant therapy, respectively. Breast adenocarcinoma (34%), lung cancers (33%), blood cancers (7%), and cancers originated from the digestive system (26%) were the greater part of cases.

The rate of the TCRFCQ-V1.0 was completed extremely high (97% of the patients completed all questions), and the missing rate was low (0.2%–1.1% for each question).

### Internal reliability

A satisfactory Cronbach alpha value should range from 0.7 to 0.9. Our Cronbach alpha value was 0.889 (Table 1). Five items were eliminated because their respective Cronbach alpha values were higher than the total mean value of Cronbach's alpha. Overall, the TCRFCQ-V1.0 had adequate Cronbach alpha coefficients (range, from 0.882 to 0.889; Table 1).

**Table 1:** Scale of the Taiwan cancer-related fatigue questionnaire verified through item and reliability analyses

Item Number	Critical ratio test (CR; i.e., *t* value)	Homogeneity test (α value of the total reliability analysis is 0.889)		Remark
		Correlation between items and total scale (R)	Cronbach’s α value	
Q1	3.763	0.320	0.888	
Q2	3.117	0.364	0.887	
Q3	4.802	0.428	0.886	
Q4	3.329	0.310	0.888	
Q5	3.373	0.329	0.888	
Q6	4.642	0.362	0.887	
Q7	3.540	0.337	0.887	
Q8	6.248	0.516	0.885	
Q9	6.108	0.500	0.886	
Q10	−0.220 (t < 3)	0.008	0.891(> 0.889)	delete
Q11	8.678	0.581	0.884	
Q12	9.362	0.675	0.883	
Q13	5.598	0.421	0.887	
Q14	2.413	0.216	0.889	
Q15	6.642	0.543	0.885	
Q16	7.674	0.548	0.885	
Q17	2.778	0.280	0.888	
Q18	2.778	0.223	0.889	
Q19	7.810	0.557	0.885	
Q20	11.960	0.681	0.883	
Q21	2.619(t < 3)	0.447	0.906(> 0.889)	delete
Q22	10.778	0.683	0.882	
Q23	10.215	0.711	0.883	
Q24	9.584	0.617	0.884	
Q25	7.119	0.507	0.886	
Q26	8.725	0.618	0.884	
Q27	13.835	0.744	0.882	
Q27	13.835	0.744	0.882	
Q28	11.462	0.715	0.882	
Q29	9.783	0.704	0.882	
Q30	7.537	0.643	0.883	
Q31	9.885	0.658	0.884	
Q32	−8.376(t < 3)	−0.586	0.899(> 0.889)	delete
Q33	8.492	0.609	0.884	
Q34	−8.019(t < 3)	−0.552	0.898(> 0.889)	delete
Q35	−0.266(t < 3)	−0.037	0.892(> 0.889)	delete
Q36	7.161	0.517	0.885	
Q37	10.269	0.591	0.884	
Q38	8.879	0.594	0.884	
Q39	5.439	0.493	0.886	
Q40	7.218	0.513	0.886	
Q41	5.459	0.448	0.886	
Q42	5.397	0.460	0.886	
Q43	4.907	0.433	0.887	

Note: The highest and lowest values were tested in the critical ratio test.

### Construct validity

The Bartlett's test in the current study were significant (chi-squared, 2390.11; *p* < 0.001) which indicated the factor analysis were appropriate. The KMO statistic of 0.868 showed the adequate sampling in the study. By using EFA, we identified 6 factors with eigenvalues of > 1. The scree plot indicated no flattening factors. We identified a six factor model as the most clinically significant for CRF cofnition. Thus, we repeat EFA, and used varimax rotation as a solution of 6 factor. Overall, a factor loading of ≥ 0.55 was achieved in 28 items. In addition, based on the high currency of not fulfilled and the clinical significance between cancer patients, 7 additional items were maintained. The factor analysis were not contributed by these 7 additional factors (Table 2). Furthermore, there were no ≥ 0.55 factor loading and ≥ 15% levels of cognition in the 7 additional factors. Consequently, we removed the 7 items considered not as clinical significance from the tool. The detailed factor loadings of the items are listed in Table 2. The TCRFCQ-V1.0 includes 6 dimensions of CRF, namely unfocused life, attribution cognition, help expectation, treatment helplessness, physician–patient communication, and life power. These 6 dimensions accounted for 67% of the total variance (Table 3). Four EFA procedures are summarized in Table 4. The findings indicate that our newly developed TCRFCQ-V1.0 is a trusty and appropriate instrument for measuring CRF cognition in Taiwanese cancer patients.

**Table 2:** Exploratory factor analysis of items of the Taiwan cancer-related fatigue questionnaire

CRF summary with the maximum variation method							
New items (re-edited ordering)	Unfocused life	Attribution cognition	Help expectation	Treatment helplessness	Physician–patient communication	Life power	Commonality
Q1	0.834	0.046	0.113	0.222	0.136	0.118	0.792
Q2	0.813	0.174	0.119	0.178	0.184	0.044	0.773
Q3	0.801	0.102	0.139	0.127	0.169	0.263	0.785
Q4	0.760	0.032	0.175	0.162	0.134	0.070	0.659
Q5	0.649	−0.008	0.086	0.290	0.118	0.326	0.633
Q6	0.613	−0.008	0.215	0.366	0.243	0.182	0.649
Q7	0.034	0.878	0.088	0.119	−0.047	0.046	0.798
Q8	0.103	0.860	0.091	0.203	−0.012	0.014	0.799
Q9	0.009	0.842	0.079	0.059	0.025	−0.014	0.720
Q10	0.080	0.680	0.028	−0.193	0.230	0.076	0.565
Q11	0.043	0.638	−0.055	−0.118	0.202	0.292	0.552
Q12	−0.009	0.073	0.837	0.102	0.194	0.013	0.754
Q13	0.056	−0.006	0.724	0.243	0.004	0.087	0.595
Q14	0.148	0.131	0.701	−0.143	0.090	0.304	0.652
Q15	0.218	0.090	0.645	0.160	0.201	−0.243	0.597
Q16	0.344	0.086	0.624	−0.127	0.131	0.166	0.575
Q17	0.177	−0.061	0.491	0.168	0.242	0.241	0.421
Q18	0.340	0.047	−0.010	0.722	0.155	0.095	0.672
Q19	0.341	0.072	0.244	0.639	0.030	0.348	0.710
Q20	0.507	0.021	0.128	0.573	0.032	0.195	0.641
Q21	0.396	0.012	0.139	0.560	0.311	0.179	0.618
Q22	0.396	0.008	0.104	0.469	0.423	0.062	0.570
Q23	0.189	0.167	0.213	−0.028	0.780	0.056	0.721
Q24	0.171	0.113	0.280	0.203	0.721	0.249	0.743
Q25	0.297	0.077	0.174	0.245	0.719	0.026	0.702
Q26	0.147	0.145	0.030	0.050	0.229	0.747	0.656
Q27	0.308	0.124	0.224	0.283	0.052	0.720	0.762
Q28	0.279	0.078	0.188	0.306	−0.020	0.650	0.636

**Table 3:** Exploratory factor analysis of 6 dimensions of the Taiwan cancer-related fatigue questionnaire

	CRF summary with the maximum variation method					
New items (re-edited ordering)	Unfocused life	Attribution cognition	Help expectation	Treatment helplessness	Physician –patient communication	Life power
Eigen values	4.764	3.271	3.27	2.68	2.433	2.336
Reserved items	6	5	6	5	3	3
Explained variance	17.013	11.682	11.666	9.572	8.689	8.344
Cumulative amount of explained variance	66.966					
Subscale Cronbach's α	0.910	0.852	0.809	0.847	0.814	0.794
Total table Cronbach's α	0.933					
Adequacy of sampling (KMO)	0.868					
Bartlett's spherical test χ^2^ value	χ^2^ ( 378, *N* = 148) = 2390.11, *p* < 0.001					

**Table 4:** Four exploratory factor analyses of the Taiwan cancer-related fatigue questionnaire

EFA times	KMO > 0.4	Aspect numbers	Numbers of deleted items	Deleted items
1	0.868	7 8	3	7 6 26
2	0.867	7	2	8 16
3	0.863	7	2	13 15
4	0.868	6	0	

## DISCUSSION

All patients with cancer should be screened for CRF in the first visit; at the finish of primary treatment, as indicated during clinical follow-up survivor care when the diagnosis of advanced disease is made; and at each therapy visit [1]. Screening should be performed and documented using a quantitative or semiquantitative assessment. One example is the visual analog scale [1, 11–13]. Other instruments have been developed and validated for quantifying CRF [14]. Some of the more commonly used and satisfactorily validated instruments are the fatigue and anemia subscales ofBrief Fatigue Inventory (BFI) [13], the Functional Assessment of Cancer Therapy instrument (FACT-F), Bidimensional Fatigue Scale (BFS) [15, 16], European Organization for Research and Treatment of Cancer QOL Questionnaire (EORTC QLQ-C30) and Multidimensional Fatigue Symptom Inventory-Short Form (MFSI-SF) [17]: fatigue subscale [18]. However, trusty and appropriate instrument for measuring fatigue and CRF cognition is essential for successful clinical care of Taiwanese patients with cancers who experience this troubling symptom. Because fatigue is subjective, clinicians must rely on patient self-reports to assess its presence and severity, which can then be supported by additional sources of information such as physical examination, laboratory data, and family members’ descriptions. In addition, the results of a questionnaire differ in different societies [19–21]. Lack of equivalent measures has been one of the difficulties encountered by researchers intending to perform cross-cultural research [20–22]. Most researchers could not ensure the equivalence of a translated measure's back translation. Although basic methods may ensure translational equivalence, the translated edition and original measures perform in the equivalent of psychometrically manners might be different [19–21]. Because of different cultures, languages, and social classes [23], developing a Taiwanese CRF cognition questionnaire is crucial to the investigation of CRF and fatigue-related cognition in the future. According to our review of the literature, the TCRFCQ-V1.0 is the first instrument for estimating CRF and fatigue-related cognition in Taiwanese patients with cancers. We suggest that in the future, all patients with cancers should be screened for CRF cognition at the initial visit, during cancer treatments, and every 3 months after treatments. Screening patients with cancers at different therapeutic intervals can be crucial to understanding the optimal treatment time for CRF in these patients.

Fatigue is a common problem in patients with cancers and occurs in patients receiving aggressive cancer therapy as well as in cancer patients who have completed all cancer treatment. Progressive cancer growing, cytotoxic CT, targeted therapy, biological response modifiers, RT or CCRT, anemia, pain, poor nutrition emotional distress, and sleep disturbance were contributed to CRF. QOL of both patients and their families were affected deeply by CRF, whatever psychosocial, physical and even occupational or economic fields [4–6, 24]. However, in contemporary oncological treatment, the main issue of supportive care are the treatment and screening of CRF. Screening for CRF in cancer patients are recommended based on American Society of Clinical Oncology and NCCN guidelines [1, 25]. If any of the factors associated with fatigue is identified, these should be addressed as an initial approach to fatigue. Optimizing the management of CRF-associated symptoms such as pain, nausea, and dyspnea can help in alleviating fatigue and further improving the QOL of both patients and their families. The QOL of patients with cancer without CRF can be improved and optimal cancer treatment can be provided if they report being out of symptoms of fatigue.

Our tool seems reliable, feasible and validity in view of the preliminary data of psychometric measurements. First, we believe that the questionnaire satisfactorily covers the sentiences of CRF expressed by cancer patients under study because no cancer patient gained the lowest or highest probable entire score and dimension score. Besides it indicates that the measure, at least theoretically, can express poor or good changes with proper tolerance in all dimensions (< 15% cancer patients have extreme score in the total score or any of the dimentions) [26]. Second, the completion rate is good (97%), which is calculated as the rate of cancer patients not missing responses in any retained question, was quite satisfactory. A group of 43 items was to evaluate CRF in cancer patients and each item was assessed in terms of importance and frequency. And there were only some items selected for cancer patients. The quantity of information lost during the performance of questionnaire would be low. The content of this type of questionnaire in Taiwanese cancer patients would be acceptable according to our results. Regarding the cause and effect (reflexivity) of the qualitative data, the TCRFCQ-V1.0 includes 6 dimensions of CRF. The naming of the dimensions was based on the original questions in similar psychological concepts. We named these dimensions on the basis of our own clinical and academic experience; thus, we believe that our study is creative and original.

As listed in Table 1, The satisfaction of Cronbach alpha coefficients were achieved. Over the suggested minimum of 0.70 of the Cronbach alpha values for all the dimensions and the total score were reached for use at the questionnaire. [27]. This type of questionnaire used in individual level should be over 0.90 in the total score [26]. This is especially crucial because developing of a Taiwanese questionnaire which could assess CRF in cancer patient was our goal. After cancer patients who answered 43 items twice, the reported values were gained within the context of the item reduction study. Cancer patients completing the final version of the questionnaire and required to respond to each of the items only once would improve the quality of our questionnaire. The reliability of the TCRFCQ-V1.0 was satisfactory in our study.

CRF is a distressing psychological condition experienced by patients with cancer, and CRF cannot be easily measured. A psychometric approach is suitable for evaluating the emotions of people. EFA, which was used in the study, is a statistical method that can reveal the basic structure of fatigue. In this study, we reviewed the literature in MEDLINE^®^, PubMed^®^, National Cancer Institute, and Chinese academic databases to design and analyze the TCRFCQ-V1.0 as well as to connect the psychological knowledge base with the clinical understanding of cancers. The feasible of factor analysis were presented well according to the significant results of Bartlett's test (chi-squared, 2390.11; *p* < 0.001; Table 3). The KMO statistic of 0.868 also implied the adequacy of sampling. All KMO statistics of EPA (Table 4) were significant. Compared with the defined groups, the known groups showed coherent between the expected range after the validity analysis. The TCRFCQ-V1.0 includes 6 dimensions of CRF, namely unfocused life, attribution cognition, help expectation, treatment helplessness, physician–patient communication, and life power. These 6 factors accounted for 67% of the total variance (Table 3). The inclusion of the 6 CRF dimensions in the TCRFCQ-V1.0 is a strength of this study. The 6 factors are different from those included in previous CRF questionnaires such as FACT-F, BFI [13], BFS [15, 16], MFSI-SF [17], and EORTC QLQ-C30: fatigue subscale [18]. The depth and breadth of the TCRFCQ-V1.0 are more extensive.

There are some limitations in the current study. Although the completion rate of the TCRFCQ-V1.0 was high (97% of the patients completed all questions) and missing ratewas low (0.2%–1.1% for each question), a small amount of misleading information may be present in our study. Furthermore, the patient sample studied may not be the deputy of the general population of Taiwanese cancer patients, because the cancer patients recruited were from a single institute in Taiwan. Moreover, 87% of the patients had a high education level (at least high school). Thus, the extrapolation of the TCRFCQ-V1.0 to patients having a low education level might be insufficient.

### Conclusions

The TCRFCQ-V1.0 has satisfactory internal consistency and reliability and is a convenient-to-use clinical measure of fatigue cognition in Taiwanese cancer patients. In the future, the prevalence of CRF and fatigue-related cognition among Taiwanese patients with cancer will be investigated. The refinement and use of the TCRFCQ-V1.0 can help in supporting CRF cognition research and clinical management, in addition to improving communication between patients and clinicians.

## MATERIALS AND METHODS

### Item generation

We reviewed the literature in MEDLINE^®^, PubMed^®^, National Cancer Institute, and Chinese academic databases to design and analyze the TCRFCQ-V1.0 as well as to connect the psychological knowledge base with the clinical understanding of cancers. The responses of the TCRFCQ-V1.0 were recorded using a 5-point response scale (strongly disagree, disagree, ordinary, agree, or very much agree). More than 90% of patients selected the lowest or highest category, and the initial 43 items in the TCRFCQ-V1.0 were thus assessed for floor and ceiling effects, respectively. Items with these effects were removed from further analysis (3 items). The final items in the TCRFCQ-V1.0 were selected on the basis of a combination of statistical evidence and clinical relevance. The total score was the summation of the retained items. Higher scores indicated higher levels of CRF cognition.

### Patients

We enrolled patients with cancer who were aged > 18 years, had received a pathological diagnosis of cancer, and had undergone cancer treatments such as surgery, chemotherapy (CT), radiotherapy (RT), and concurrent chemoradiotherapy (CCRT) at a single institute in Taiwan. Our protocols were reviewed and approved by the Institutional Review Board of Taipei City Hospital (TCHIRB-1021103-E). The enrolled patients were asked to complete the 43-item, investigator-designed TCRFCQ-V1.0 (See Supplementary Table 1 for the version translated into English). The survey was conducted by trained study nurses in the outpatient department of a single institute. After informed consent was obtained from patients, they were enrolled in the study.

### Questionnaire

The TCRFCQ-V1.0 (the initial 43-item version) was verbally delivered by trained interviewers and was completed through personal interviews for all patients (100%). The interviewers received standardized training, and interviews were monitored for consistency across study sites. The patients were provided a hard copy of response categories, and the interviewer recorded their responses. Although the mentioned tools have not been specifically tested, they have been extensively validated within the Taiwan population. Additionally, these tools were used during the early stages of TCRFCQ-V1.0 development, and preliminary evidence determines them to be suitable with respect to content and language.

### Statistical analysis

Statistical analyses were conducted using SPSS software (Version 20; IBM Corporation, Armonk, NY). We used descriptive statistics to summarize the demographic and clinical characteristics of the patients and the prevalence of CRF cognition. Furthermore, we used Bartlett's test of sphericity and the Kaiser–Meyer–Olkin (KMO) measure of sampling adequacy to examine the appropriateness of the sample size for conducting exploratory factor analysis (EFA). Factors with eigenvalues of > 1 were identified using principal component analysis, and scree plots were used to determine the point at which the decrease in eigenvalues became negligible. A 6-factor model was selected because it was the most clinically meaningful model. Furthermore, we conducted EFA, forcing a 6-factor solution with varimax rotation. The conventional primary factor loading cutoff value of ≥ 0.55 was used to identify items for retention, which were then attributed to the factor with the highest loading. Items within identified factors were assessed for their internal consistency by using Cronbach's alpha reliability coefficients. Items for which the patients reported ≥ 15% moderate to high levels of cognition but had loadings that were lower than the cutoff value were retained due to clinical significance. The convergent validity of the total cognition score was tested using the Spearman rank-order correlation and the Pearson correlation coefficient. Discriminant validity was assessed by comparing median CRF scores by using the Mann–Whitney or Kruskal–Wallis test.

## SUPPLEMENTARY MATERIALS FIGURES AND TABLES




