# Supplementary material for: Development of a Taiwan cancer-related fatigue cognition questionnaire: reliability and validity
Source: Oncotarget. 2017 Mar 16;8(17):28880–7. doi: 10.18632/oncotarget.16285 (PMC5438699; doi:10.18632/oncotarget.16285)
Supplement: Supplementary file 1 [file oncotarget-08-28880-s001.pdf]

Development of a Taiwan cancer-related fatigue cognition questionnaire: reliability and validity

Supplementary Materials

Supplementary Table 1: Details of the Taiwan cancer-related fatigue questionnaire, version 1.0.
See_Supplementary_Table 1
