# Supplementary material for: Development of a Taiwan cancer-related fatigue cognition questionnaire: reliability and validity
Source: Oncotarget. 2017 Mar 16;8(17):28880–7. doi: 10.18632/oncotarget.16285 (PMC5438699; doi:10.18632/oncotarget.16285)
Supplement: Supplementary file 2 [file oncotarget-08-28880-s002.docx]

**Taiwan Cancer-Related Fatigue Questionnaire, Version 1.0**

**Basic Information**

Sex □ Male □ Female

Age

Height

Body weight

Education level

Marital status □ Single □ Married

Occupation

Do you have any income? □ Yes □ No

□ NT$10000–NT$20000 □ NT$21000–NT$30000 □ NT$31000–NT$40000

□ NT$41000–NT$50000 □ NT$51000–NT$60000 □ > NT$60000

**Treatment Stage**

- Untreated □ Undergoing treatment □ Completion of treatment

**Therapeutic Class**

- Chemotherapy □ Radiotherapy □ CCRT

**Questionnaire**

|  | **very much agree** | **agree** | **ordinary** | **disagree** | **strongly disagree** |
| --- | --- | --- | --- | --- | --- |
| **Unfocused life** |  |  |  |  |  |
| I feel that I become sensitive and suspicious |  |  |  |  |  |
| I feel lonely and helpless |  |  |  |  |  |
| My lifestyle has become disordered |  |  |  |  |  |
| My social activity has decreased |  |  |  |  |  |
| The tiredness makes me think negatively |  |  |  |  |  |
| I feel difficulty in paying attention |  |  |  |  |  |
| **Attribution cognition** | | | | | |
| I think fatigue is caused by chemotherapy and radiotherapy |  |  |  |  |  |
| I think fatigue is a symptom caused by targeted therapy |  |  |  |  |  |
| I think fatigue is a symptom caused by surgery and cancer |  |  |  |  |  |
| I think anemia may cause fatigue |  |  |  |  |  |
| I think anxiety or depression may cause fatigue |  |  |  |  |  |
| **Help expectation** |  |  |  |  |  |
| I want to receive professional nutrition guidance |  |  |  |  |  |
| I want to receive music therapy to relieve pressure |  |  |  |  |  |
| I hope that the problem of tiredness can be solved |  |  |  |  |  |
| I hope that I can talk with a professional psychologist or a social worker |  |  |  |  |  |
| I hope to receive adjuvant therapy of traditional medicine |  |  |  |  |  |
| I hope that I can be taken care of with dignity |  |  |  |  |  |

**Questionnaire (continue)**

|  | **very much agree** | **agree** | **ordinary** | **disagree** | **strongly disagree** |
| --- | --- | --- | --- | --- | --- |
| **Treatment helplessness** |  |  |  |  |  |
| I have no confidence in the completion of treatment |  |  |  |  |  |
| Fatigue makes me anxious |  |  |  |  |  |
| I often feel angry |  |  |  |  |  |
| When severe fatigue occurs, I do not want to receive treatment |  |  |  |  |  |
| I am afraid of being looked at |  |  |  |  |  |
| **Physician–patient communication** | | | | | |
| I am afraid that the doctor may give up on me |  |  |  |  |  |
| I am worried that I cannot get the most appropriate treatment |  |  |  |  |  |
| I worried that the issue I raise will upset doctors |  |  |  |  |  |
| **Life power** |  |  |  |  |  |
| I still feel tired even after waking up |  |  |  |  |  |
| I feel lazy such that I do not want to move |  |  |  |  |  |
| I get tired after housework that I used to do easily |  |  |  |  |  |
